# Surgical Techniques for a Parasitic Myoma Fed by the Mesentery: A Case Report and Technical Discussion

**DOI:** 10.7759/cureus.100968

**Published:** 2026-01-06

**Authors:** Yu Horibe, Miho Matsuda, Akira Nakabayashi, Jun Kumakiri, Tsutomu Tabata

**Affiliations:** 1 Gynecology, Tokyo Women's Medical University, Tokyo, JPN; 2 Obstetrics and Gynecology, Tokyo Women's Medical University, Tokyo, JPN

**Keywords:** laparoscopic surgery, mesentery, parasitic leiomyoma, parasitic myoma, surgical technique guide

## Abstract

Parasitic myoma (PM) is a rare form of leiomyoma that detaches from the uterus and acquires a blood supply from other organs. While often iatrogenic following power morcellation, primary spontaneous PM can occur. We present a surgical case of a PM fed by the mesentery, likely resulting from inflammatory adhesions following a previous small bowel ileus. A 33-year-old nulliparous woman with no history of prior surgeries underwent laparoscopic myomectomy for a suspected subserosal myoma. Intraoperatively, we encountered a PM with a significant vascular supply extending from the mesentery to the uterine wall. The tumor was firmly adherent to the gastrointestinal tract. We examined the technical management of high-risk vascular adhesions. Key maneuvers included: (1) injection of vasopressin strictly on the uterine side of the attachment to avoid compromising mesenteric blood flow; (2) precise identification of the dissection plane; and (3) meticulous dissection alternating between monopolar excision and blunt separation to preserve the bowel integrity. The tumor was successfully detached, and the feeding vessels were coagulated. A containment bag was used during morcellation to prevent iatrogenic seeding. Operative time was 145 minutes with no bowel injury.

This case highlights that PM should be considered even in patients without prior gynecologic surgery if there is a history of intra-abdominal inflammation. Careful identification of dissection planes and management of feeding vessels are critical for safe laparoscopic management.

## Introduction

Parasitic myoma (PM) is a rare type of leiomyoma that lacks direct attachment to the uterus and instead receives its blood supply from other organs. The overall incidence of PM is estimated to be between 0.12% and 0.95% [1]. It is a rare entity, particularly when involving the small intestine, which is reported to account for only 3.3% (5/151 cases) of such cases [2]. Although generally benign, PMs can undergo sarcomatous transformation. The reported malignant transformation rate of 2%-5% in PMs is notably higher than that observed in intrauterine leiomyomas, which is approximately 0.2%-0.3% [3].

While there are various theories regarding its pathogenesis, two dominant hypotheses exist: primary (or spontaneous) PM, in which a pedunculated subserosal myoma detaches from the uterus for some reason and reattaches to other pelvic organs; and secondary (or iatrogenic) PM, in which myoma fragments scattered during surgery remain in the abdominal cavity and subsequently implant and grow. Preoperative evaluation of vascularity is essential in PM cases, as the blood supply originates from organs other than the uterus. Utilizing dynamic CT and 3D-CT angiography alongside MRI facilitates the identification and visual mapping of abnormal feeding vessels, including the omental, superior vesical, and inferior mesenteric arteries, thereby reducing the risks of intraoperative bleeding and visceral injury [4]. The choice of surgical approach - laparoscopic, open, or, more recently, robot-assisted surgery - is determined based on tumor size, anatomical location, and suspicion of malignancy. In particular, robot-assisted surgery offers significant advantages in performing precise maneuvers for tumors situated in complex regions, such as the deep pelvis and the retroperitoneum [5,6].

In this report, we present a case of PM incidentally encountered during surgery, involving a patient who had previously developed a small bowel ileus. This case illustrates the surgical challenges posed by a mesenteric blood supply and discusses techniques for safe dissection. It is hypothesized that the tumor acquired a blood supply from the mesentery. This report details a rare case of PM with a high-risk mesenteric blood supply, highlighting critical surgical techniques for safe dissection.

## Case presentation

**Figure 1:**
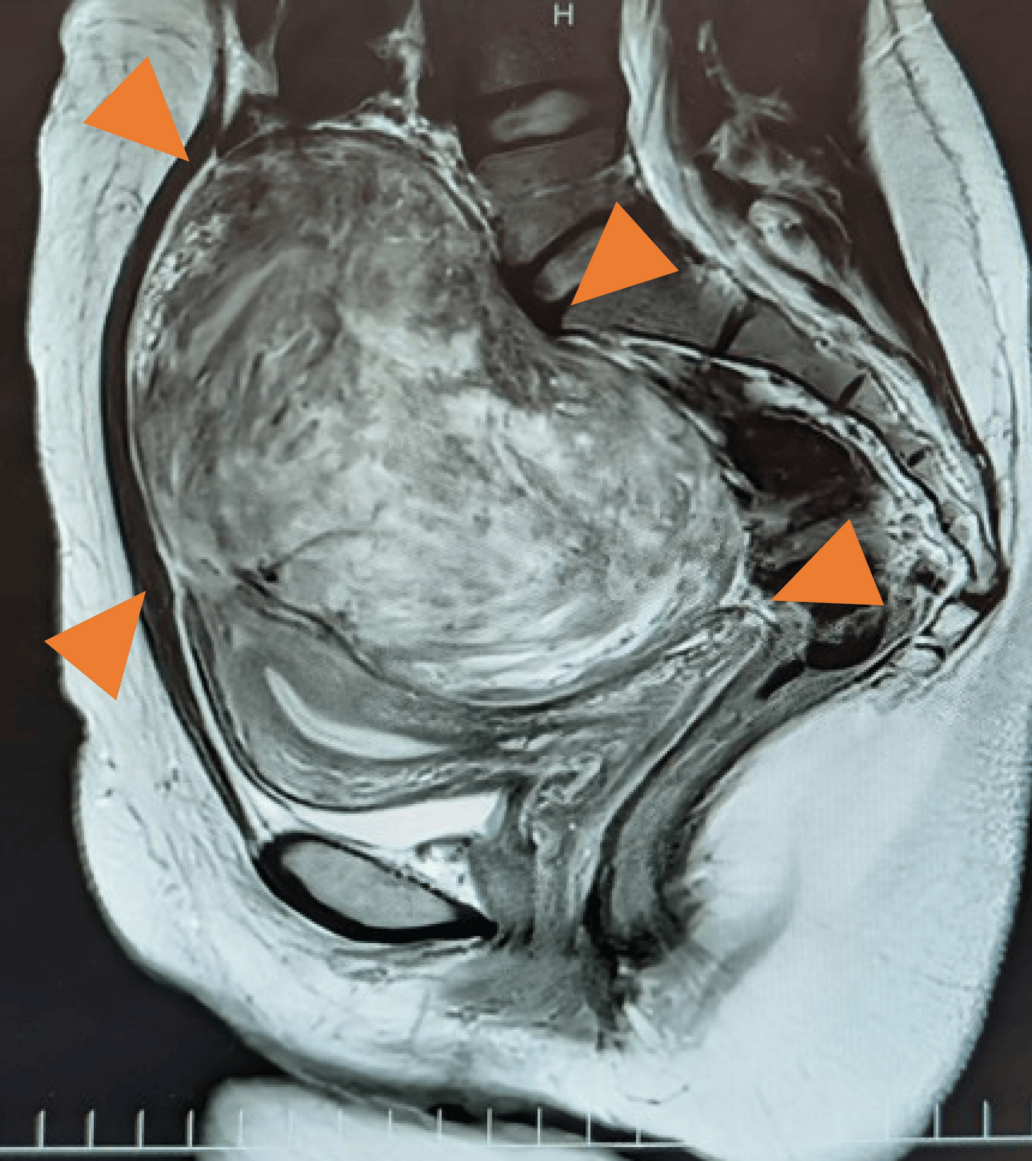
MRI findings of subserosal fibroids The image shows a subserosal fibroid arising from the fundus of the uterus (arrowheads), but no flow void to the mesentery or small bowel could be confirmed.

The patient was a 33-year-old female with no history of pregnancy (gravida 0, para 0), comorbidities, or prior surgeries. Six months prior to the laparoscopic surgery at our hospital, she developed a small bowel ileus and was admitted to another hospital for emergency care. At that time, a subserosal uterine myoma was identified, and she was referred to our Gynecology Department (Figure 1). Although the serum lactate dehydrogenase (LDH) level was elevated at 293 U/L, imaging studies revealed a subserosal uterine leiomyoma with no findings suggestive of malignancy.

Following administration of gonadotropin-releasing hormone antagonist (GnRH), relugolix 40 mg per day, the plan was to proceed with a laparoscopic myomectomy.

During the surgery, preoperative MRI findings suggested a single pedunculated subserous fibroid, but intraoperative findings revealed that the pedunculated subserous fibroid was a PM that had developed feeding vessels not only from the uterus but also into the mesentery.

Video 1 presents the intraoperative findings and a discussion of the surgical techniques employed.

**Video 1:** Laparoscopic parasitic fibroid surgery This report describes the surgical maneuvers and technical precautions for an incidentally discovered parasitic myoma.

First, we accessed the abdominal cavity using a parallel approach and performed an intra-abdominal observation. We confirmed that adhesions from the feeding vessels extended to the peritoneum of the Douglas fossa. To minimize the risk of compromising blood flow to the gastrointestinal tract, vasopressin was injected into the uterine side, where the feeding vessels of the mesentery are attached. Subsequently, we proceeded with meticulous dissection, alternating between monopolar excision and blunt dissection, starting from the uterine side. This technique allowed for successful separation without any injury to the gastrointestinal tract. An additional vascular supply was identified from the pedicle of the subserosal myoma, which was then coagulated and transected.

The fundamental surgical approach involves coagulating the feeding vessels to the fibroid to minimize intraoperative bleeding. This is followed by careful dissection of the fibroid from the surrounding tissue to prevent damage to adjacent organs. In this particular case, however, extensive adhesions to the gastrointestinal tract significantly elevated the risk of iatrogenic injury. Consequently, it is preferable to perform resection closer to the uterus than to the feeding vessels to minimize damage to the gastrointestinal tract.

**Figure 2:**
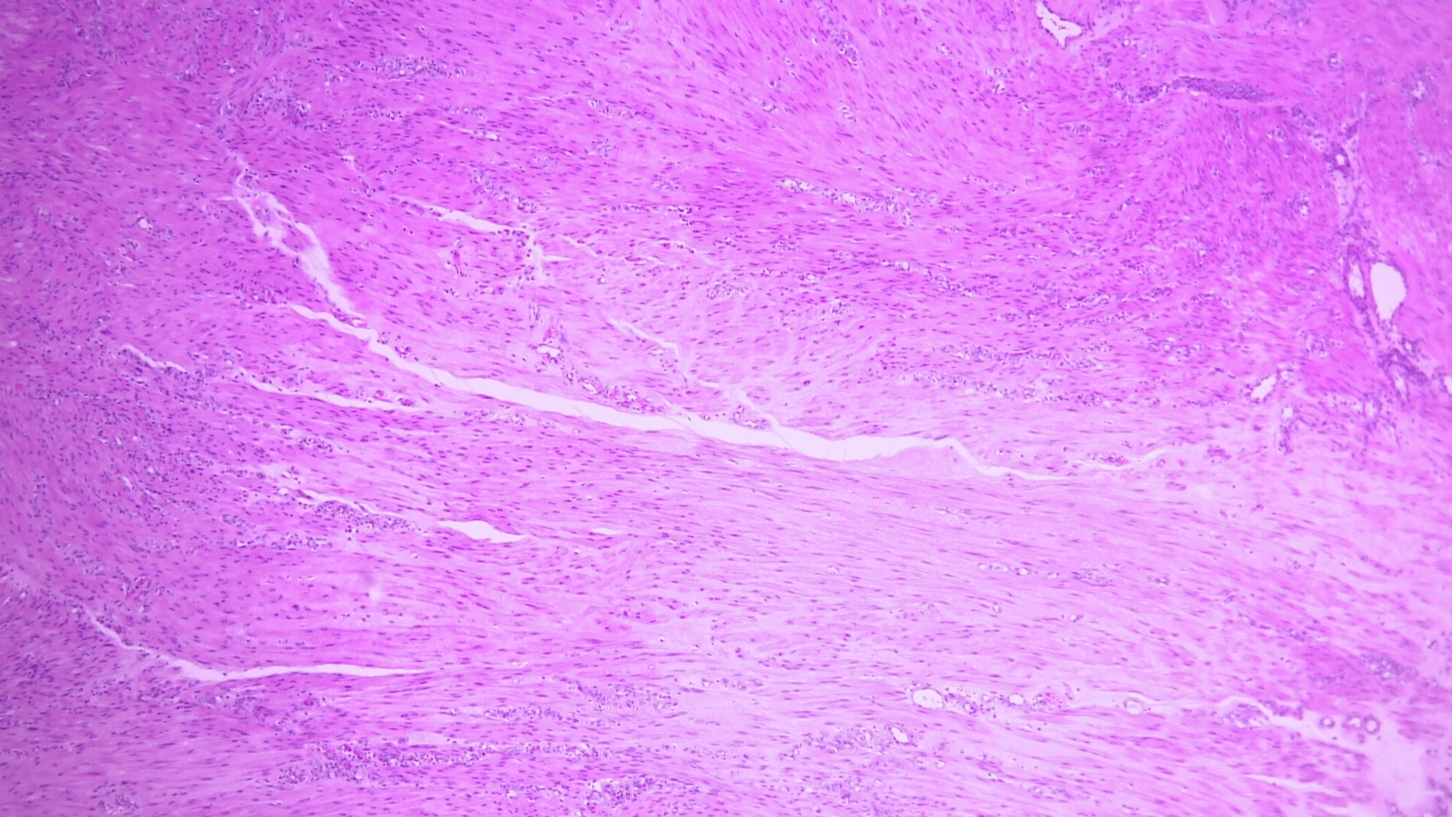
Microscopic findings Microscopic findings reveal interlacing fascicles of spindle cells with cigar-shaped nuclei and eosinophilic cytoplasm. Nuclear atypia is minimal. These findings are consistent with leiomyoma.

In this case, a significant portion of the operative time was dedicated to identifying the correct surgical plane and performing the dissection. The total operative time was 145 minutes, with an estimated blood loss of 400 mL. The resected specimen weighed 506 g, and the final pathological diagnosis was leiomyoma (Figure 2).

The patient’s postoperative course was uneventful, and she was discharged four days after the surgery. To date, 15 months postoperatively, there has been no evidence of uterine fibroid recurrence.

The postoperative course was uneventful, with no complications such as anemia or infection. The patient was discharged on postoperative day 4.

## Discussion

PM is a rare leiomyoma that lacks direct attachment to the uterus and develops by receiving a blood supply from other organs. The International Federation of Gynecology and Obstetrics (FIGO) classifies uterine myomas into eight types, with PM classified as Type 8 (non-myometrial location) [7].

Regarding its pathogenesis, several theories exist, with the following being the most prevalent: (1) primary/spontaneous PM, a historically recognized type in which a pedunculated subserosal myoma separates from the uterus due to torsion or other reasons, adheres to other pelvic organs, and continues to grow [3,8]; (2) secondary/iatrogenic PM, a type in which myoma fragments scattered during surgery (such as morcellation) remain in the abdominal cavity, implant, and grow [3]; and (3) nonsurgical iatrogenic PM, in which blood flow to the uterus is restricted due to GnRH agonist administration or uterine artery embolization (UAE), causing the myoma to separate from the uterus and become parasitic by obtaining blood flow from other organs [8].

While the typical implantation sites for primary or spontaneous PM include the omentum and peritoneum [2], the present case involved a subserosal uterine myoma nourished by blood vessels from both the mesentery and the uterus. Given the patient’s lack of prior gynecological surgery, the exact incidence of such a presentation is unknown [9]. Consequently, this case was considered a variant of primary or spontaneous PM.

This double-vascularized pedunculated serosa myoma was considered to be a pedunculated subserosal fibroid with a secondary parasitic blood supply rather than a true PM classified by FIGO Type 8, in which the tumor was acquiring a parasitic blood supply via mesenteric adhesions induced by inflammation associated with the patient’s history of bowel obstruction and peritonitis.

Alternatively, another mechanism is that small intestinal ileus developed because the pedunculated serosa myoma originally spread through the feeding vessels into the gastrointestinal tract. These are hypotheses rather than a proven causal mechanism, so it is necessary to accumulate case studies.

Although the incidence of PM following laparoscopic morcellation is reported to be 0.12%-0.95% [1], making it a relatively recognized entity, the preoperative diagnosis of PM in this spontaneous case proved to be difficult.

Regarding treatment, surgical resection is considered the standard approach. While laparoscopic surgery is feasible and effective in many cases, laparotomy is indicated in cases where laparoscopy is unsuitable due to tumor size or location, or when malignancy is suspected [5]. Alternatively, conservative management may be an option for elderly patients, those with multiple comorbidities, or in asymptomatic cases where the tumor is strongly suspected to be benign [3]. Finally, similar to uterine leiomyomas, PMs often exhibit positivity for estrogen and progesterone receptors; therefore, hormonal therapy may be effective as a pharmacological treatment [10].

Generally, because PM is often discovered incidentally intraoperatively and involves a wide variety of parasitic sites, there is no standardized surgical technique. However, successful management relies on meticulous intra-abdominal observation, gentle dissection from adhered organs [11], and secure ligation of feeding vessels. Furthermore, to prevent the development of new iatrogenic PM during specimen retrieval, it is crucial to perform morcellation within a containment bag [12].

## Conclusions

When planning surgery for uterine myomas, if there is a history of myomectomy or UAE, or if imaging suggests the presence of PM, a specific surgical strategy can be formulated preoperatively. However, it is also crucial to plan for the possibility that the tumor may have formed feeding vessels with surrounding organs due to intra-abdominal inflammation, even in the absence of surgical history. In the present case, the patient had no prior history of myomectomy, which is typically considered a significant factor in the development of PM. Furthermore, it is of paramount importance to preoperatively rule out malignancy when diagnosing PM. We sincerely hope that this video will serve as a useful reference for laparoscopic surgery for PM.
